# Investigating factors influencing quality of life in thyroid eye disease: insight from machine learning approaches

**DOI:** 10.1530/ETJ-24-0292

**Published:** 2025-01-09

**Authors:** Haiyang Zhang, Shuo Wu, Lehan Yang, Chengjing Fan, Huifang Chen, Hui Wang, Tianyi Zhu, Yinwei Li, Jing Sun, Xuefei Song, Huifang Zhou, Terry J Smith, Xianqun Fan

**Affiliations:** ^1^ Department of Ophthalmology, Shanghai Ninth People’s Hospital, Shanghai Key Laboratory of Orbital Diseases and Ocular Oncology, and Center for Basic Medical Research and Innovation in Visual System Diseases of Ministry of Education, Shanghai Jiao Tong University School of Medicine, Shanghai, China; ^2^ Nursing Department, Shanghai Ninth People’s Hospital, Shanghai Jiao Tong University School of Medicine, Shanghai, China; ^3^ Department of Ophthalmology and Visual Sciences and Department of Internal Medicine, Kellogg Eye Center-Michigan Medicine and University of Michigan, Ann Arbor, Michigan, USA

**Keywords:** Thyroid eye disease, quality of life, machine learning, Shapley additive explanations (SHAP), XGBoost

## Abstract

**Aims:**

Thyroid eye disease (TED) is an autoimmune orbital disorder that diminishes the quality of life (QOL) in affected individuals. Graves’ ophthalmopathy (GO)-QOL questionnaire effectively assesses TED’s effect on patients. This study aims to investigate the factors influencing visual functioning (QOL-VF) and physical appearance (QOL-AP) scores in Chinese TED patients using innovative data analysis methods.

**Methods:**

This cross-sectional study included 211 TED patients whose initial visit to our clinic was from July 2022 to March 2023. Patients with previous ophthalmic surgery or concurrent severe diseases were excluded. GO-QOL questionnaires, detailed medical histories and clinical examinations were collected. The distribution of GO-QOL scores was analyzed, and linear regression and machine learning algorithms were utilized.

**Results:**

The median QOL-VF and QOL-AP scores were 64.29 and 62.5, respectively. Multivariate linear regression analysis revealed age (*P* = 0.013), ocular motility pain (*P* = 0.012), vertical strabismus (*P* < 0.001) and diplopia scores as significant predictors for QOL-VF. For QOL-AP, gender (*P* = 0.013) and clinical activity (*P* = 0.086) were significant. The XGBoost model demonstrated superior performance, with an *R*^2^ of 0.872 and a root mean square error of 11.083. Shapley additive explanations (SHAP) analysis highlighted the importance of vertical strabismus, diplopia score and age in influencing QOL-VF and age, clinical activity and sex in QOL-AP.

**Conclusion:**

TED significantly affects patient QOL. The study highlights the efficacy of XGBoost and SHAP analyses in identifying key factors influencing the QOL in TED patients. Identifying effective interventions and considering specific demographic characteristics are essential to improving the QOL of patients with TED.

## Introduction

Thyroid eye disease (TED) is an autoimmune orbital disorder and the most important extrathyroidal manifestation of Graves’ disease (GD) (1). Approximately 50% of patients with GD develop TED, and these patients often suffer from eyelid redness and swelling, pain, proptosis, ocular motility restriction, diplopia and, in some cases, visual deterioration. These persistent signs and symptoms seriously affect the quality of life (QOL).

A person’s QOL reflects their well-being and ability to function in life, influenced by factors such as vision loss, financial strain and social relationships (2). Assessing TED merely through physical examination often underestimates its effect (3). To better evaluate the QOL in TED patients, a Graves’ ophthalmopathy (GO)-QOL questionnaire, developed by Terwee and coworkers (4), is commonly used. This questionnaire consists of 16 questions focusing on two specific aspects: eight items assessing visual functioning (QOL-VF) and eight items addressing physical appearance (QOL-AP). Understanding the effect of TED on patient well-being is crucial for identifying factors that affect QOL, thus enabling the development of new strategies to improve disease management (5).

The QOL in TED patients can be influenced by many factors, which can offer valuable insight for treating the disease. However, results from earlier studies relying on the GO-QOL questionnaire have varied because of factors such as small sample sizes, data quality issues and differences in analytical methods (6, 7, 8). Consensus regarding the relative importance of factors such as sex, age, activity level and disease severity has yet to be achieved. To better understand the factors affecting GO-QOL scores, more comprehensive clinical data are needed.

Machine learning (ML) is a sophisticated computational method used for analyzing data, handling missing values and tackling overfitting and underfitting issues (9, 10). ML, often accompanied by Shapley additive explanations (SHAP), a method for identifying the characteristics of indicators in prediction models, has been employed in the analysis of factors influencing the QOL (11, 12). Previous studies using GO-QOL have predominantly relied on univariate and multivariate analyses. ML emerges as an underutilized yet promising method for predicting GO-QOL scores in patients with TED. Therefore, we embarked on implementing ML algorithms for regression modeling and SHAP for feature analysis of the QOL in TED patients.

The current study aimed to better understand the factors influencing the QOL in patients with TED, using a Chinese population as an example. A questionnaire survey was provided to TED patients visiting our clinics. Detailed clinical data of the patients were collected and studied, employing various ML models to identify and interpret the factors influencing the QOL.

## Methods

### Patients

This retrospective study was approved by the ethics committee (see the ‘Ethics approval consent to participate’ section for details). TED patients visiting the outpatient clinic at our department of ophthalmology for the first time from July 2022 to March 2023 were recruited for this study. The inclusion criteria were as follows: (i) TED diagnosis according to the European Group on Graves’ Orbitopathy (EUGOGO) clinical guidelines at our outpatient clinic (13); (ii) aged between 18 and 75 years; and (iii) completion of the GO-QOL self-administered questionnaires at the outpatient clinic before undergoing examination. The exclusion criteria were as follows: (i) previous orbital decompression surgery or strabismus surgery; (ii) presence of systemic comorbidities such as diabetes, hepatitis or other ocular diseases such as cataracts and glaucoma, etc.; and (iii) lack of key medical history or clinical examination records. In total, 67 subjects were excluded (17, 29 and 21 for the three exclusion criteria).

### GO-QOL questionnaires

The QOL in TED patients was assessed using the GO-QOL questionnaire, which was routinely administered before appointments. A small number of patients (less than 30 individuals) declined to complete the questionnaire because of time constraints or other reasons. The questionnaire, sourced from the EUGOGO website, was translated into Chinese and comprised two subscales: QOL-VF and QOL-AP, with each item rated on a three-point scale (1 = severely limited, 2 = a little limited and 3 = not limited at all). Researchers conducted face-to-face interactions to explain, administer and record the questionnaire. Scores ranged from 8 to 24, which were then converted to a scale of 0–100. In this conversion, 0 represented the worst QOL and 100 represented the best. The formula used for conversion was as follows: (total scores − *X*)/(2 × *X*) × 100, where *X* meant the number of completed items (4). QOL-VF and QOL-AP scores for each patient were obtained as response variables.

### Demographic and clinical profiles

To assess the factors affecting patient QOL, we conducted a retrospective analysis, gathering demographic and clinical data from medical records, including sex, age, education and disease duration since the first TED-related ocular signs or symptoms. Treatment histories such as intravenous steroids and other interventions (radiotherapy, local injection, oral steroids and immunosuppressants) were noted. We also evaluated TED activity using the clinical activity score (CAS) and orbital MRI results. Patients with a CAS ≥ 3 or noticeable inflammatory changes on orbital MRI were categorized as having active TED, while those without these criteria were classified as inactive TED (14). The severity was determined using the EUGOGO classification system, dividing patients into mild, moderate-to-severe and sight-threatening categories.

Patient clinical profiles, such as best-corrected visual acuity, palpebral fissure height and proptosis as measured by Hertel exophthalmometry, were collected by a single ophthalmologist (15). The thyroid status was considered stable if serum-free thyroxine (FT4), serum free triiodothyronine (FT3) and thyroid stimulating hormone levels were all within the normal range. Recorded periocular inflammatory signs included five objective signs of CAS: eyelid redness, eyelid swelling, conjunctival redness, conjunctival swelling and caruncle or plica swelling. Ocular motility restriction was considered positive if the patient experienced difficulty in moving the eye in any direction. Strabismus was measured by prism cover testing in different gaze directions. Those with a horizontal deviation greater than 15 PD or a vertical deviation greater than 10 PD were considered to have strabismus (16). The Gorman subjective diplopia score was also evaluated, which ranges from 0 to 3: no diplopia (0), intermittent diplopia in primary gaze when awakening or tired (1), diplopia at extremes of gaze (2) and continuous diplopia in primary or reading gaze (3). Additionally, patients were asked about symptoms, including spontaneous retrobulbar pain, ocular motility pain, photophobia, excessive tearing and dry eyes, with responses recorded as yes or no.

### Predictive modeling with machine learning algorithms

We individually implemented various machine learning models, including ensemble methods such as XGBoost (eXtreme Gradient Boosting), AdaBoost (adaptive boosting), random forest and gradient boosting; decision-based algorithms such as decision tree; and regularization techniques such as ridge, lasso regression and elastic net (17, 18, 19, 20). Additionally, we utilized support vector regression, K-nearest neighbors (K-neighbors) and multilayer perceptron regression (MLP regression) to address different predictive challenges (21). According to an 8:2 ratio, the 211 patients were randomly divided into a training group and a verification group, consisting of 169 and 42 patients, respectively.

To analyze the contributions of each feature in the optimal prediction model, we utilized the SHAP methodology through the ‘SHAP’ Python library. SHAP values were computed to illustrate how each variable influences the model’s output (22). By using the ‘SHAP. Explainer’ function, we fitted the model and derived SHAP values, which gauge the expected change in the model output per feature. These values facilitated the creation of a feature importance plot, demonstrating the magnitude and direction of each feature’s effect on the predictions. This process helped identify key features that significantly affect outcomes. For a more detailed explanation of these machine learning algorithms in predictive modeling, see the Supplementary Method (see the section on Supplementary materials given at the end of the article).

### Statistical analysis

The baseline characteristics of TED patients were outlined using descriptive statistics and frequency tables. Indicators were categorized based on the variables under study. Categorical variables were summarized using frequency tables. Numerical variables following a normal distribution were presented as mean ± standard deviation (SD), while those deviating from normality were described using the interquartile range and median. Spearman rank correlation or Pearson correlation analysis was employed to assess the correlation between numerical indicators and the GO-QOL score, while the Mann–Whitney test was utilized for categorical indicators. For potential influencing factors of the GO-QOL score identified in the univariate analysis, multivariate linear regression was conducted to evaluate the effect of each influencing factor. Statistical significance was set at *P* < 0.05. The analyses were conducted using SPSS 19.0.

To assess the predictive capabilities of our models, we utilized several statistical metrics, including the mean absolute error, mean square error (MSE), *R*^2^, variance and root MSE (RMSE). This facilitated a comprehensive comparison between the ML algorithms and the conventional statistical approach of linear regression.

## Results

### Characteristics of participants

The demographic and clinical characteristics of the study participants are shown in Table 1. Among the participants, 138 cases (65.4%) were female, with a mean age at study entry of 43.6 ± 13.2 years. The median duration of TED was 12 months, ranging from 6 to 26 months. Approximately equal proportions of TED patients exhibited stable (51.2%) and unstable (48.8%) thyroid activity statuses. The majority of patients had not previously received intravenous steroids (151 cases, 71.6%) or other TED-related treatments (165 cases, 78.2%, including oral steroids, local steroid injections, immunosuppressants and radiotherapy). Within the cohort, 100 patients (47.4%) had active TED. Most patients (144 cases, 68.3%) were diagnosed with moderate-to-severe TED. The most frequently reported periocular inflammatory sign was eyelid swelling (136 cases, 64.5%). Common symptoms included photophobia (60.2%) and excess tearing (58.8%). Additionally, 58.8% of patients presented with varying degrees of diplopia, with constant diplopia accounting for 37% of cases, representing the most prevalent category.

**Table 1:** Demographic and clinical characteristics of patients with TED. Data are presented as *n* (%) or mean ± SD or median (Q1, Q3).

Characteristics	Values
Sex	
Male	73 (34.6)
Female	138 (65.4)
Age (years)	43.6 ± 13.2
Education (years)	15 (9,16)
Disease duration (months)	12 (6, 26)
Thyroid status	
Stable	108 (51.2)
Unstable	103 (48.8)
TED treatment history	
Intravenous steroids	60 (28.4)
Other treatment	46 (21.8)
Activity	
Inactive	111 (52.6)
Active	100 (47.4)
Severity	
Mild	25 (11.8)
Moderate-to-severe	144 (68.3)
Sight-threatening	42 (19.9)
Best-corrected visual acuity	1.0 (0.8,1.0)
Proptosis (mm)	19.50 (18.00, 21.25)
Palpebral fissure height (mm)	10.0 (8.5,11.0)
Periocular inflammatory signs	
Eyelid redness	14 (6.6)
Eyelid swelling	136 (64.5)
Conjunctival redness	61 (28.9)
Conjunctival swelling	59 (28.0)
Caruncle or plica swelling	71 (33.6)
Discomfort symptoms	
Spontaneous retrobulbar pain	57 (27.0)
Ocular motility pain	37 (17.5)
Photophobia	127 (60.2)
Excess tearing eyes	124 (58.8)
Dry eyes	89 (42.2)
Ocular motility restriction	148 (70.1)
Strabismus	
Vertical strabismus	58 (27.5)
Horizontal strabismus	66 (31.3)
Diplopia score	
0: no diplopia	87 (41.2)
1: intermittent diplopia	8 (3.8)
2: gaze-dependent diplopia	38 (18.0)
3: constant diplopia	78 (37)

TED, thyroid eye disease.

### QOL impairment in TED patients

**Figure 1:**
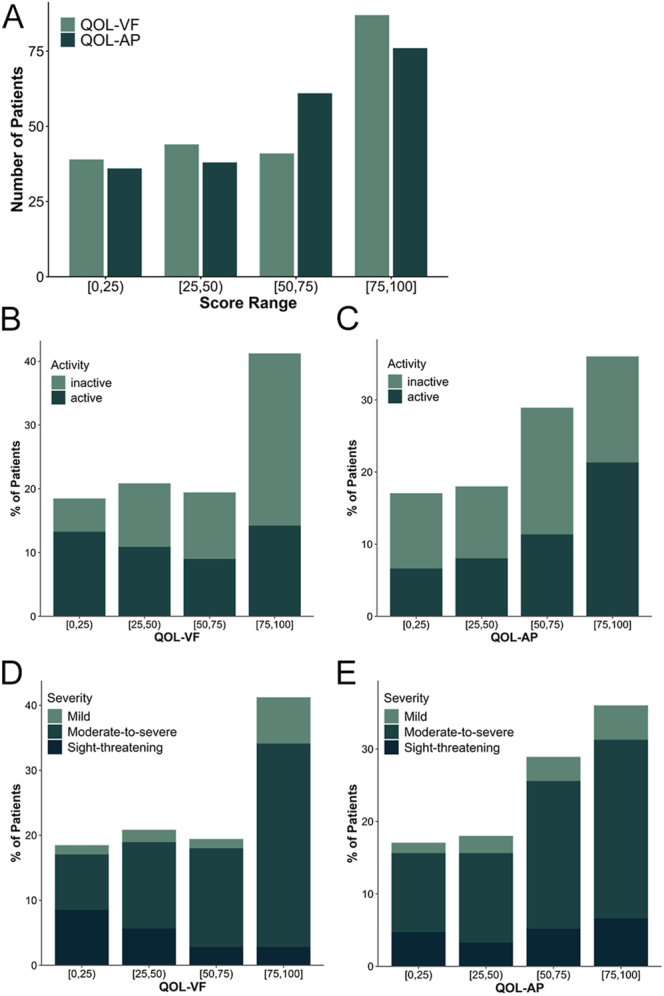
Distribution of QOL-VF and QOL-AP among patients with TED. (A) Distribution of QOL-VF and QOL-AP scores. Score ranges are divided into (0–25), (25–50), (50–75) and (75–100). Distribution of QOL-VF (B) and QOL-AP (C) scores in relation to activity levels (active vs inactive). Distribution of QOL-VF (D) and QOL-AP (E) scores across disease severities (mild, moderate-to-severe and sight-threatening). QOL, quality of life; QOL-VF, visual functioning; QOL-AP, appearance.

Patient QOL was affected to varying extents by TED (Fig. 1A). The median QOL-VF score stood at 64.29 (42.86, 93.75), with 41.23% (87 patients) achieving scores above 75, indicating relatively good QOL-VF. Regarding QOL-AP, the median score was 62.5 (43.75, 87.50), with 36.02% (76 patients) scoring above 75 and 13.27% (28 patients) falling below 25. Additionally, 11 patients scored below 25 in both categories, while 35 patients scored above 75 in both. In Fig. 1B, inactive patients dominate the highest QOL-VF score category (75, 100). Conversely, Fig. 1C reveals a higher proportion of active patients in the uppermost QOL-AP score range. In Fig. 1D and E, a greater percentage of patients with mild conditions fall within the (75–100) range for both VF and AP, whereas those with sight-threatening disease are most frequently found in the (0–25) score range. In summary, most patients experienced mild to moderate impairment in their QOL scores.

### Results of the univariate and multivariate analysis

#### Visual functioning subscale

Univariate analyses disclosed that 17 out of 26 variables were significantly associated with QOL-VF (Table 2). Female patients exhibited higher QOL-VF scores (66.76 ± 31.65) compared with their male counterparts (52.16 ± 33.65, *P* = 0.002). QOL-VF scores showed a negative correlation with age (*P* < 0.001) and a positive correlation with years of education (*P* = 0.008) and disease duration (*P* = 0.002). Patients who previously underwent intravenous steroids (*P* < 0.001) or other treatments (*P* = 0.007) had significantly lower QOL-VF scores.

**Table 2:** Results of univariate analysis.

	QOL-VF			QOL-AP		
	Mean ± SD	t	*P*	Mean ± SD	t	*P*
Sex			0.002*		2.77	0.006*
Male	52.16 ± 33.65	−3.11		69.18 ± 27.15		
Female	66.76 ± 31.65			57.74 ± 29.25		
Age (years)		−6.76	<0.001*	0.128	1.5	0.134
Education (years)		2.69	0.008*	0.002	−0.07	0.945
Disease duration (months)		3.18	0.002*	−0.097	0.82	0.412
Thyroid status		1.46	0.145		0.05	0.957
Stable	64.96 ± 32.91			61.81 ± 29.85		
Unstable	58.31 ± 33.01			61.59 ± 28.22		
Intravenous steroids		−4.87	<0.001*		−1.8	0.074
Yes	45.00 ± 31.01			56.04 ± 35.73		
No	68.35 ± 31.54			63.95 ± 25.63		
Other treatment		−2.74	0.007*		−1.37	0.171
Yes	50.08 ± 35.55			56.52 ± 36.42		
No	64.96 ± 31.68			63.14 ± 26.51		
Activity		−4.53	<0.001*		2.09	0.038*
Inactive	71.06 ± 28.57			57.77 ± 28.58		
Active	51.33 ± 34.69			66.06 ± 28.97		
Severity				−0.07		0.313
Mild	75.77 ± 31.87			65.75 ± 28.02		
Moderate–severe	66.91 ± 30.19	−1.35	0.179	62.07 ± 28.69	−0.59	0.559
Sight-threatening	35.52 ± 30.04	−5.25	<0.001*	58.04 ± 30.84	−1.05	0.294
Best-corrected visual acuity		6	<0.001*	0.132	1.4	0.163
Proptosis (mm)		1.08	0.282	−0.063	−0.17	0.864
Palpebral fissure height (mm)		1.93	0.055	0.028	0.77	0.441
Eyelid redness		0	0.998		0.88	0.379
Yes	61.69 ± 31.38			68.30 ± 29.86		
No	61.71 ± 33.24			61.23 ± 28.95		
Eyelid swelling		−2.45	0.015*		0.01	0.99
Yes	57.62 ± 32.40			61.72 ± 29.82		
No	69.13 ± 33.14			61.67 ± 27 .63		
Conjunctival redness		−1.47	0.144		0.22	0.824
Yes	56.49 ± 32.80			62.40 ± 30.16		
No	63.84 ± 33.02			61.42 ± 28.61		
Conjunctival swelling		−2.16	0.032*		0.02	0.133
Yes	53.90 ± 32.73			61.76 ± 32.85		
No	64.75 ± 32.78			61.68 ± 27.47		
Caruncle or plica swelling		−1.82	0.071		−1.51	0.184
Yes	55.94 ± 32.15			57.48 ± 30.45		
No	64.64 ± 33.23			63.84 ± 28.10		
Spontaneous retrobulbar pain		−2.46	0.015*		−0.49	0.624
Yes	52.61 ± 33.56			60.09 ± 31.78		
No	65.08 ± 32.32			62.30 ± 27.98		
Ocular motility pain		−3.47	<0.001*		−1.03	0.307
Yes	45.01 ± 30.16			57.26 ± 32.56		
No	65.26 ± 32.62			62.64 ± 28.19		
Photophobia		−1.88	0.061		−1.02	0.307
Yes	58.25 ± 33.91			60.04 ± 29.41		
No	66.95 ± 31.18			64.21 ± 28.34		
Excess tearing		−0.99	0.325		0.3	0.767
Yes	59.83 ± 34.05			62.20 ± 28.89		
No	64.39 ± 31.57			60.99 ± 29.30		
Dry eyes		−1.64	0.103		0.79	0.429
Yes	57.37 ± 33.55			63.55 ± 27.57		
No	64.88 ± 32.45			60.35 ± 30.03		
Ocular motility restriction		−3.99	<0.001*		−0.33	0.745
Yes	55.98 ± 32.82			61.28 ± 30.42		
No	75.17 ± 29.72			62.70 ± 25.55		
Vertical strabismus		−9.2	<0.001*		−0.05	0.958
Yes	32.96 ± 28.87			61.53 ± 30.25		
No	72.61 ± 27.59			61.76 ± 28.60		
Horizontal strabismus		−4.27	<0.001*		0.69	0.494
Yes	47.86 ± 32.89			63.73 ± 26.16		
No	68.02 ± 31.25			60.78 ± 30.24		
Diplopia score	−0.47			0.022		0.752
0: no diplopia	80.07 ± 26.01			61.14 ± 27.93		
1: intermittent diplopia	35.94 ± 29.30	−4.11	<0.001*	64.83 ± 32.03	0.34	0.731
2: gaze-dependent diplopia	57.01 ± 31.72	−4.08	<0.001*	61.68 ± 30.18	0.1	0.924
3: constant diplopia	46.17 ± 30.93	−7.48	<0.001*	62.02 ± 30.18	0.19	0.846

QOL, quality of life; QOL-AP, appearance; QOL-VF, visual functioning.

* Statistical significance (*P* < 0.05).

Patients with inactive disease had a higher mean QOL-VF score (71.06 ± 28.57) compared with those with active disease (51.33 ± 34.69, *P* < 0.001). A decreasing trend in QOL-VF subscale scores was observed as TED severity progressed from mild to sight-threatening TED (*P* < 0.001). These QOL-VF scores correlated with eyelid (*P* = 0.015) and conjunctival swelling (*P* = 0.032). Objective ocular signs such as best-corrected visual acuity, ocular motility restriction, vertical strabismus, horizontal strabismus and diplopia score were significantly correlated with QOL-VF scores (*P* < 0.001). Additionally, spontaneous retrobulbar pain (*P* = 0.015) and ocular motility pain (*P* < 0.001) were also found to be significantly correlated with QOL-VF scores.

Multivariate analysis of QOL-VF scores was conducted using a linear regression model (*R*^2^ = 0.4729). The results revealed a significant predictive value for age (*P* = 0.013), ocular motility pain (*P* = 0.012), vertical strabismus (*P* < 0.001), diplopia scores, including intermittent diplopia (*P* < 0.001), gaze-dependent diplopia (*P* = 0.029) and constant diplopia (*P* = 0.024) (Table 3). According to the definitions suggested by Bradley and coworkers, there was a significant ceiling effect in the assessment of QOL-VF (40/211 = 18.96% > 15%) (23).

**Table 3:** Multivariate analysis of the predictors of quality of life-visual functioning^†^.

	Regression coefficient	Standard error	*t*	*P*
Male	−0.17	3.78	−0.05	0.963
Age	−0.39	0.16	−2.51	0.013*
Education (years)	−0.06	0.44	−0.14	0.889
Disease duration (months)	0.07	0.04	1.8	0.073
Activity	−1.02	4.31	−0.24	0.813
Severity				
Mild				
Moderate–severe	−7.44	5.68	−1.31	0.191
Sight-threatening	−11.82	9.49	−1.25	0.215
Intravenous steroids	−5.99	4.23	−1.41	0.159
Other treatment	1.36	4.35	0.31	0.756
Best-corrected visual acuity	27.24	14.07	1.94	0.054
Eyelid swelling	3.81	4.09	0.93	0.353
Conjunctiva swelling	1.07	4.38	0.24	0.807
Spontaneous retrobulbar pain	−4.39	4.34	−1.01	0.313
Ocular motility pain	−13.06	5.17	−2.53	0.012*
Ocular motility restriction	−0.26	4.2	−0.06	0.95
Vertical strabismus	−24.58	4.63	−5.31	<0.001*
Horizontal strabismus	−0.48	4.09	−0.12	0.907
Diplopia score				
0: no diplopia				
1: intermittent diplopia	−33.69	9.61	−3.5	<0.001*
2: gaze-dependent diplopia	−11.07	5.03	−2.2	0.029*
3: constant diplopia	−10.66	4.7	−2.27	0.024*

* Statistical significance (*P* < 0.05).

† Multiple linear regression model: *n* = 211, *R*^2^ = 0.4729.

#### Appearance subscale

QOL-AP scores were found to correlate with sex (*P* = 0.006) and disease activity (*P* = 0.038) (Table 2). Male patients achieved a significantly higher QOL-AP score (69.18 ± 27.15) compared with females (57.74 ± 29.25). Patients with active disease exhibited higher mean QOL-AP scores (66.06 ± 2.90) compared with inactive patients (57.77 ± 2.71). Although no significant correlation was observed between QOL-AP scores and disease severity (*P* = 0.313), a decreasing trend was noted as TED severity increased from mild to sight-threatening TED.

Multivariate analysis of QOL-AP scores was conducted using a linear regression model. The results revealed that sex, but not disease activity, represented the variable with predictive value for QOL-AP (*P* = 0.013) (Table 4). No ceiling effect was detected in the assessment of QOL-AP scores.

**Table 4:** Multivariate analysis of the predictors of quality of life-appearance^†^.

	Regression coefficient	Standard error	*t*	*P*
Male	10.37	4.16	2.5	0.013*
Activity	6.83	3.96	1.73	0.086

* Statistical significance (*P* < 0.05).

† Multiple linear regression model: *n* = 211, *R*^2^ = 0.03981.

### Evaluating the efficacy of predictive models built with machine learning algorithms

The performance metrics varied across the models, with *R*^2^ values ranging from −1.17 to 0.872 and RMSE values from 11.083 to 44.552, as detailed in Supplementary Tables 1 and 2. XGBoost emerged as the optimal model, showcasing leading performance metrics (*R*^2^ = 0.872, RMSE = 11.083) (Supplementary Tables 1 and 2). Significant variability was detected in the effectiveness of other models. For instance, multiple linear regression and AdaBoost demonstrated moderate performance, whereas decision tree and MLP regression were less effective.

### Important features predicting GO-QOL investigated by SHAP

**Figure 2:**
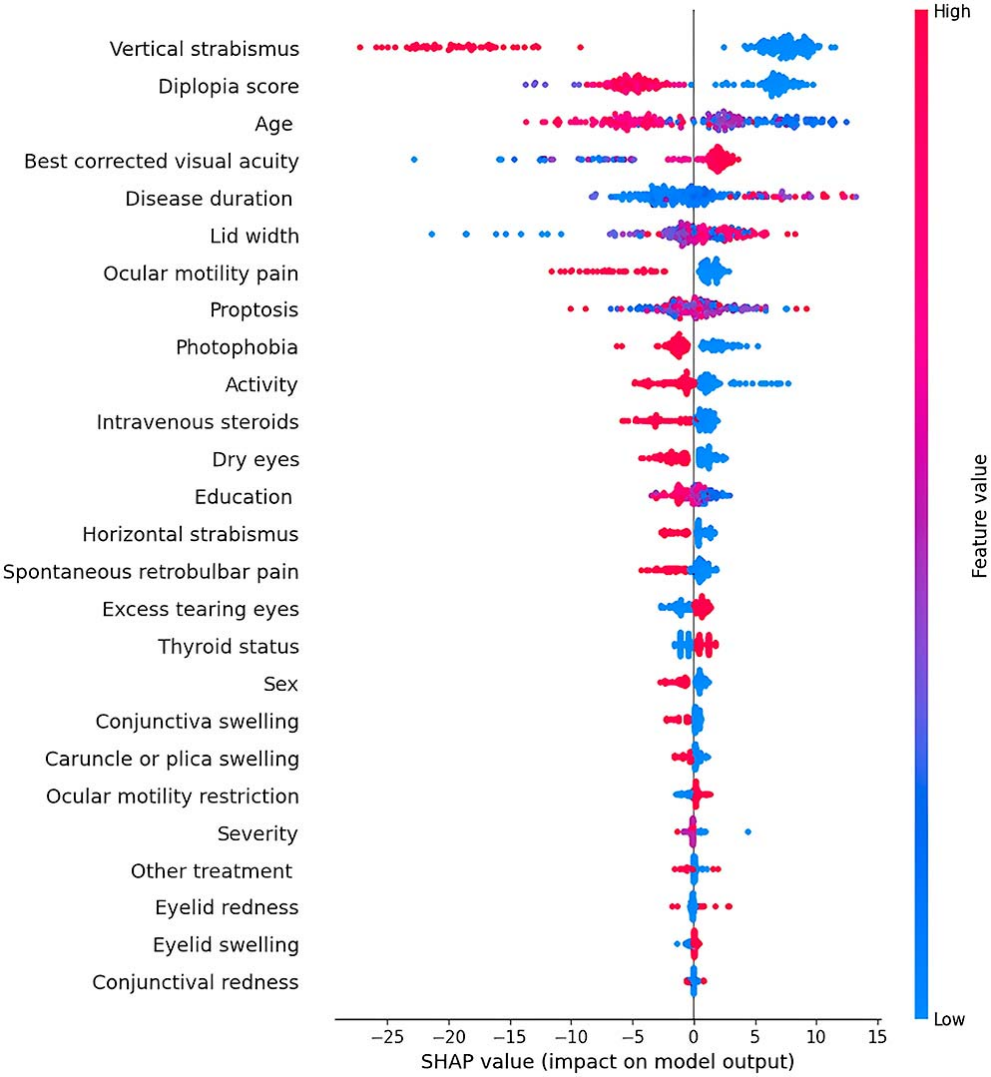
SHAP summary plot of feature importance in predicting QOL-VF. Each point represents a patient, with colors indicating feature values (red = high value and blue = low value). The *x*-axis shows SHAP values, where positive values (right) indicate features increasing QOL-AP scores and negative values (left) indicate features decreasing QOL-AP scores. Features are ordered by their absolute effect on model predictions, with the most influential features at the top. SHAP, Shapley additive explanations.

**Figure 3:**
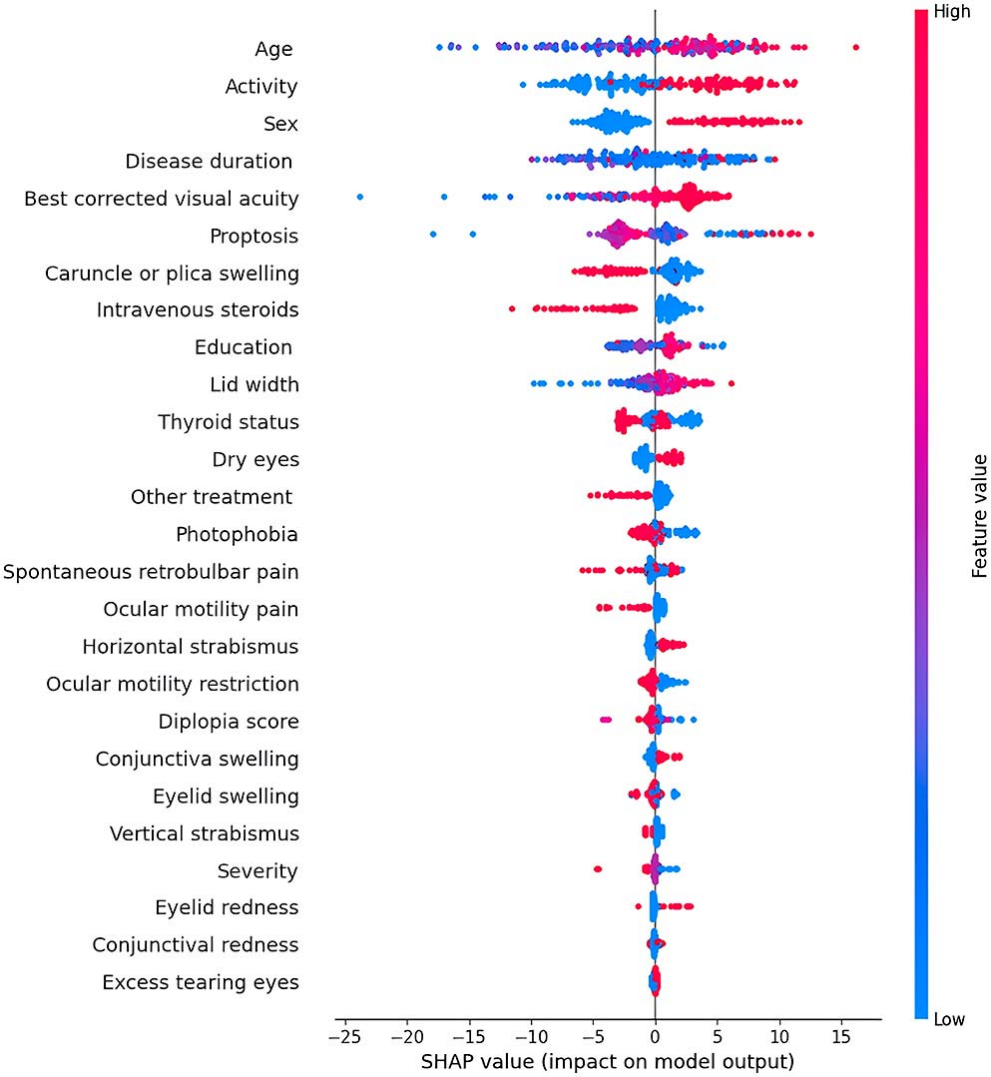
SHAP summary plot of feature importance to predict QOL-AP. Each point represents a patient, with colors indicating feature values (red = high value and blue = low value). The *x*-axis shows SHAP values, where positive values (right) indicate features increasing QOL-AP scores and negative values (left) indicate features decreasing QOL-AP scores. Features are ordered by their absolute effect on model predictions, with the most influential features at the top. SHAP, Shapley additive explanations.

The beeswarm plots generated from the SHAP analysis, illustrating the ranking of feature importance for all 26 features within the XGBoost model, are depicted in Figs 2 and 3. These plots highlight the direction of impact and provide patient-specific information, including outliers.

#### Visual functioning subscale

Vertical strabismus, diplopia score, age, best-corrected visual acuity, palpebral fissure height and ocular motility pain significantly affected the visual functioning subscale model. The SHAP analysis revealed that lower values of vertical strabismus, diplopia score and age were associated with higher positive SHAP values, indicating a positive influence on the predicted QOL-VF scores. Conversely, higher values of these features were associated with larger negative SHAP values, indicating a negative effect on the predicted QOL-VF scores (see Fig. 2). Specifically, the absence of vertical strabismus, lower diplopia scores or younger age was associated with improved vision-related QOL as predicted by the model.

#### Appearance subscale

Age, disease activity, sex, disease duration, best-corrected visual acuity and proptosis had the greatest effects on the appearance subscale model. Among these variables, disease activity and sex demonstrated a trend in which higher values corresponded to higher SHAP values, indicating increased predicted QOL-AP scores (refer to Fig. 3). Patients with active disease and male patients both showed an improvement in appearance-related QOL.

## Discussion

This study detected varying degrees of QOL reduction among the investigated population of TED patients, a finding consistent with earlier studies. QOL-VF and QOL-AP scores were lower in TED patients compared with normative values. The factors influencing the QOL in TED patients included those deemed nonmodifiable, such as sex, age and disease duration, as well as modifiable factors, including best-corrected visual acuity, ocular motility pain, vertical strabismus and diplopia.

The study revealed that patients in the active phase experienced worse QOL-VF, while those in the inactive phase were more likely to have worse QOL-AP. These findings might result from persistent appearance changes in longer-duration TED, leading to psychological stress and social challenges. This finding suggests that patients with TED, as a chronic disease, require follow-up and evaluation beyond the active period (24). We observed a statistically significant decline in QOL-VF in sight-threatening disease but detected a progressive decline in the three patient groups categorized by severity. This trend was consistent with those reported by Zeng *et al.* (25). Overall, disease severity remains a significant factor in determining QOL-VF scores in TED patients.

Sex and age significantly affect both QOL-VF and QOL-AP. Women typically score lower on QOL-AP, while men are more affected in terms of QOL-VF. Societal expectations often place more emphasis on women’s appearance, increasing their concern about their looks (26). Conversely, men might experience lower QOL-VF because of more severe ocular symptoms and a worse prognosis (27). Additionally, QOL-VF tends to decrease with age, highlighting the importance of age-appropriate treatment options to improve visual function and self-care, particularly for the elderly.

SHAP analysis demonstrated considerable agreement (i.e., a negative correlation with QOL-VF) with multivariate linear regression results, underscoring the profound effect of vertical strabismus and diplopia on QOL-VF. Diplopia could make daily activities such as reading and driving challenging and increase the risk of injuries such as falls, especially in elderly patients (28). Similarly, the study by Ponto *et al.* (29) demonstrated a significant association between diplopia and reduced QOL-VF, which aligns with our findings. McBain *et al.* (30) assessed the QOL among 220 patients with strabismus, finding scores to be markedly lower than those of the healthy cohort. Our study further indicates a more pronounced effect from vertical strabismus. This stronger association may be because of the smaller vertical fusion range and potentially greater effect on stereopsis and depth perception. Vertical deviations might also be more noticeable in social interactions. However, these hypotheses require further research. All in all, our findings suggest the potential value of strabismus correction surgery in stable TED patients for improving their QOL.

Subjective discomfort symptoms reported by patients, particularly spontaneous retrobulbar pain and ocular motility pain, correlated with a reduced QOL-VF. This correlation aligns with earlier studies, which demonstrated that an increase in ocular pain significantly diminishes the QOL (2). It underscores the importance of acknowledging the disparities that exist between patients’ and physicians’ perspectives (31) and emphasizes the potential negative effect these disparities may have on therapeutic outcomes and doctor–patient communication.

Similar to the findings of Wang *et al.* (32), patients who never received intravenous steroids or other treatments had better QOL-VF, seemingly contradicting the expected benefits of these therapies. This unexpected outcome may be attributed to several factors: patients receiving treatment likely have more severe conditions; treatment may increase patients’ psychological stress and expectations; and the complex interplay between variables could explain why treatment history was not a significant predictor in multiple linear regression analysis. These findings underscore the need for a nuanced understanding of the effect of TED treatments on patient-reported outcomes.

Earlier studies largely utilized univariate and multivariate linear regression statistical analyses (33, 34). To our knowledge, this study validates, for the first time, the factors affecting the QOL in TED patients, utilizing machine learning. Accurately interpreting ML prediction models and intuitively presenting the results to clinicians have always been a challenge. Therefore, we applied SHAP values to XGBoost to achieve optimal predictive performance and interpretability. SHAP values assess the importance of all feature combinations on the output, providing consistent and locally accurate attribution values for each feature, enabling clinicians to more intuitively understand the decision-making process of this black-box model. In addition, this model presents several unique advantages over traditional linear models. For instance, in multivariate linear regression analysis, disease duration and best-corrected visual acuity were not significantly correlated with QOL-VF; however, the results of machine learning analysis demonstrated their influence on QOL-VF. This result suggests that linear regression methods failed to capture nonlinear relationships (35).

Several limitations affect our study. First, employing a cross-sectional design identifies factors affecting a particular TED patient’s QOL at only one time point, failing to establish symptom causality. Follow-up and prospective research remain essential for evaluating the longitudinal validity of the GO-QOL. Second, the current study sampled exclusively at a single institution with a referral patient population; thus, these results may not accurately represent the broader patient population. Larger, multicenter studies will be required for validation. Finally, our research can accommodate additional parameters as potential factors influencing the QOL in TED, such as tobacco smoking habits.

Our findings highlight the potential of ML approaches in analyzing GO-QOL, opening new avenues for future research. Longitudinal studies tracking the QOL before and after treatment could provide valuable insights into the efficacy of various interventions, particularly for chronic TED patients. Furthermore, this approach could be instrumental in evaluating emerging therapies, helping to tailor treatments to individual patient needs and potentially improving overall patient outcomes. Future research should also focus on validating these findings in larger, diverse cohorts and exploring the long-term implications of QOL changes in TED management.

## Conclusion

The effect of TED on the QOL is highly variable, highlighting the need for physicians to better understand and tailor treatments based on patient-specific factors such as sex, age and disease duration. The effect of specific manifestations such as diplopia, visual acuity, pain and mobility restriction must be explored in greater detail. This study demonstrates the effectiveness of machine learning, especially XGBoost and SHAP, in analyzing QOL influences in TED.

## Supplementary materials



## Declaration of interest

The authors declare that there is no conflict of interest that could be perceived as prejudicing the impartiality of the work reported.

## Funding

This work was supported by the National Natural Science Foundation of Chinahttps://doi.org/10.13039/100017053 (82388101, 81930024 and 82271122), the Science and Technology Commission of Shanghaihttps://doi.org/10.13039/501100003399 (20DZ2270800), Shanghai Key Clinical Specialty, Shanghai Eye Disease Research Center (2022ZZ01003), and the Project of Hospital Management (YGA202303) from Shanghai Ninth People’s Hospital, Shanghai Jiao Tong University School of Medicine.

## Author contribution statement

H Zhang and S Wu conceived and designed the study, performed data analysis and interpretation and wrote the manuscript. L Yang, C Fan, H Chen, H Wang, T Zhu, Y Li, J Sun and X Song contributed to data acquisition, analysis and interpretation. H Zhou, T J Smith and X Fan provided guidance throughout the study, supervising the research and critically revising the manuscript for important intellectual content.

## Data Availability

Data sharing is not applicable to this article as no datasets were generated or analyzed during the current study.

## Ethics approval consent to participate

This retrospective study was approved by the Ethics Committee of Shanghai Ninth People’s Hospital. Given the retrospective nature of this study, the ethics committee approved the waiver of individual informed consent.
