# Supplementary material for: Investigating factors influencing quality of life in thyroid eye disease: insight from machine learning approaches
Source: Eur Thyroid J. 2025 Jan 9;14(1):e240292. doi: 10.1530/ETJ-24-0292 (PMC11816044; doi:10.1530/ETJ-24-0292)
Supplement: Supplementary file 1 [file supplementary_materials.pdf]

Supplementary Material

Suppl. Table 1. Performance of models of QOL-VF built with machine learning algorithms.

	R^2	RMSE	MSE	MAE	VARIANCE
XGBoost	0.872	11.083	122.831	8.313	
Adaboost	0.6546	18.186	330.734	15.931	
Ridge Regression	0.22	27.332	747.015	21.797	674.911
Lasso Regression	0.299	25.913	671.467	20.428	495.108
Elastic Net	0.228	27.18	738.778	21.63	412.176
Decision Tree	0.161	28.344	28.3442	21.207	1337.714
Random Forest	0.347	25.008	625.383	19.527	526.215
Gradient Boosting	0.101	29.334	860.492	22.717	683.831
SVR	0.058	30.036	902.147	25.47	23.428
KNeighbors	0.045	30.245	914.789	25.688	422.46
MLP Regression	0.207	27.557	759.391	21.797	637.312

Supplementary Material

Suppl. Table 2. Performance of models of QOL-AP built with machine learning algorithms.

	R^2	RMSE	MSE	MAE	VARIANCE
XGBoost	0.793	13.768	189.544	11.261	
Adaboost	0.509	21.172	448.254	18.784	
Ridge Regression	-0.269	34.069	1160.72	28.791	201.982
Lasso Regression	-0.104	31.77	1009.317	26.902	44.288
Elastic Net	-0.077	31.388	985.229	26.398	14.738
Decision Tree	-1.17	44.552	1984.92	35.32	744.828
Random Forest	-0.15	32.43	1051.702	27.513	59.621
Gradient Boosting	-0.299	34.463	1187.719	28.381	162.873
SVR	-0.048	30.966	958.896	25.672	1.53
KNeighbors	-0.038	30.806	948.983	27.151	72.423
MLP Regression	-0.275	34.147	1166.046	29.429	231.93

Supplementary Method

Appendix: Machine Learning Algorithms in Predictive Modeling

XGBoost is an ensemble learning algorithm based on decision trees that improves prediction accuracy by iteratively constructing multiple weak learners. Its core principle involves using gradient boosting to build new decision trees in each iteration to correct residuals from previous models. XGBoost assumes the presence of learnable complex patterns in the data, including non-linear relationships and feature interactions [1]. Unlike linear regression, XGBoost automatically captures these complex relationships without the need to prespecify interaction terms. Regarding data processing, XGBoost can handle various data types, including numerical and categorical, is insensitive to feature scaling, and can automatically manage missing values [1].

AdaBoost (Adaptive Boosting) is an ensemble learning method that combines multiple weak learners to form a strong predictive model[2]. The core idea behind AdaBoost is to iteratively adjust the weight of training instances, giving more focus to those that were misclassified in previous iterations. By emphasizing harder-to-classify instances, AdaBoost increases the model's ability to correct errors and improve overall performance. Unlike XGBoost, which uses gradient boosting, AdaBoost uses a weighted average of weak learners, typically decision trees with a limited depth, to form the final prediction.

Random Forest is an ensemble method that constructs a collection of decision trees during training and outputs the mode or mean prediction of the individual trees[3]. The algorithm operates by creating trees using random subsets of the data and features, which reduces overfitting and increases model generalization. Random Forest is effective in handling large datasets with numerous features and can manage both regression and classification tasks. Its ability to perform feature selection implicitly by averaging over many trees makes it robust to noise and high-dimensional data.

Gradient Boosting builds an additive model by sequentially fitting new models to the residuals (errors) of the combined predictions of the previous models[4]. Each subsequent model aims to reduce the overall prediction error by correcting the mistakes made by prior models. Unlike AdaBoost, which assigns weights to individual data points, Gradient Boosting adjusts the predictions by fitting new models to the errors in the residuals. This method is widely used for its high accuracy but is prone to overfitting if not carefully tuned.

Decision Tree is a non-linear model used for both classification and regression tasks[5]. It partitions the feature space into distinct regions based on simple decision rules, which are learned from the data. The decision tree algorithm recursively splits the data based on feature values that result in the best splits according to a chosen criterion, such as Gini impurity or mean squared error. Although decision trees are interpretable and simple, they tend to overfit if the tree is too deep or lacks proper pruning.

Ridge Regression is a linear regression method that incorporates L2 regularization to address multicollinearity or overfitting in the model[6]. The regularization term adds a penalty to the size of the coefficients, shrinking them towards zero but not forcing them to be exactly zero. This helps

to stabilize the estimates when the predictor variables are highly correlated. Ridge regression is particularly effective in situations with many predictors and can improve model generalization by reducing variance.

Lasso Regression (Least Absolute Shrinkage and Selection Operator) is another linear regression technique that applies L1 regularization[6]. Unlike Ridge, which only shrinks coefficients, Lasso can force some coefficients to exactly zero, effectively performing feature selection. This makes Lasso useful for models with a large number of predictors, where automatic selection of the most relevant features is necessary. Lasso enhances model interpretability and can improve performance when many features are irrelevant or redundant.

Elastic Net combines the penalties of both Ridge and Lasso regression[7]. By incorporating both L1 and L2 regularization terms, Elastic Net is effective in situations where there are multiple correlated features. It benefits from the stability of Ridge and the sparsity of Lasso, making it particularly useful when the number of predictors is larger than the number of observations. Elastic Net allows for a balance between feature selection and coefficient shrinkage, resulting in a more robust model.

Support Vector Regression (SVR) is a regression technique based on the concept of Support Vector Machines (SVM)[8]. SVR attempts to fit a hyperplane in a high-dimensional feature space while minimizing the prediction error within a specified margin. The algorithm focuses on instances that are within a margin of error, ignoring those that lie outside of the margin. SVR is particularly effective when the relationship between features and target variables is highly non-linear, as it uses kernel functions to map the data into higher dimensions.

K-Nearest Neighbors (K-NN) is a simple, non-parametric algorithm that makes predictions based on the nearest neighbors in the feature space[9]. For regression tasks, the prediction is the average of the target values of the K nearest neighbors. K-NN does not make explicit assumptions about the data distribution and is particularly effective in capturing complex, non-linear relationships. However, it can be computationally expensive, especially with large datasets, and sensitive to the choice of K and the distance metric used.

Multilayer Perceptron Regression (MLP Regression) is a type of artificial neural network used for regression tasks[10]. It consists of multiple layers of interconnected neurons that model complex relationships between input features and the target variable. MLP Regression is capable of learning non-linear mappings and can be highly flexible, depending on the network architecture and activation functions. However, MLP models require careful tuning of hyperparameters such as the number of layers, neurons per layer, and learning rate to avoid overfitting and ensure effective training.

SHAP is a method for interpreting machine learning model predictions, based on the concept of Shapley values from game theory [11]. The fundamental principle of SHAP is to view each feature as a "player" and the prediction outcome as the "game's payoff," calculating the marginal contribution of each feature to the prediction result. SHAP assumes potential interactions between features but does not require specific distribution assumptions for the data, making it particularly suitable for analyzing complex clinical data [12–14].

Machine learning models offer several significant improvements over traditional statistical

methods such as linear regression. Firstly, they can capture non-linear relationships and complex interactions in the data, which is particularly valuable for analyzing the intricate clinical data [12–14]. Secondly, they can automatically perform feature selection and assess feature importance, reducing human bias. Moreover, these models typically exhibit higher predictive accuracy, especially when dealing with numerous features and complex data patterns.

References:

1 Chen T, Guestrin C. XGBoost: A Scalable Tree Boosting System. In: Chen T, Guestrin C. Proceedings of the 22nd ACM SIGKDD International Conference on Knowledge Discovery and Data Mining. San Francisco California USA: ACM; 2016; pp 785–94.

2 A SA, S BR, A N, J BM, H P, M BM, Uk W. Application of machine learning techniques for predicting survival in ovarian cancer. BMC medical informatics and decision making. 2022 Dec;22(1).

3 Chen Z, He N, Huang Y, Qin WT, Liu X, Li L. Integration of A Deep Learning Classifier with A Random Forest Approach for Predicting Malonylation Sites. Genomics, Proteomics & Bioinformatics. 2019 Jan;16(6):451.

4 Seto H, Oyama A, Kitora S, Toki H, Yamamoto R, Kotoku J, Haga A, Shinzawa M, Yamakawa M, Fukui S, Moriyama T. Gradient boosting decision tree becomes more reliable than logistic regression in predicting probability for diabetes with big data. Sci Rep. 2022 Oct;12:15889.

5 Wang L, Zhu L, Jiang J, Wang L, Ni W. Decision tree analysis for evaluating disease activity in patients with rheumatoid arthritis. J Int Med Res. 2021 Oct;49(10):03000605211053232.

6 Chowdhury MZI, Leung AA, Walker RL, Sikdar KC, O'Beirne M, Quan H, Turin TC. A comparison of machine learning algorithms and traditional regression-based statistical modeling for predicting hypertension incidence in a Canadian population. Sci Rep. 2023 Jan;13(1):13.

7 Ebrahimi V, Sharifi M, Mousavi-Roknabadi RS, Sadegh R, Khademian MH, Moghadami M, Dehbozorgi A. Predictive determinants of overall survival among re-infected COVID-19 patients using the elastic-net regularized Cox proportional hazards model: a machine-learning algorithm. BMC Public Health. 2022 Jan;22:10.

8 Huang J-C, Tsai Y-C, Wu P-Y, Lien Y-H, Chien C-Y, Kuo C-F, Hung J-F, Chen S-C, Kuo C-H. Predictive modeling of blood pressure during hemodialysis: a comparison of linear model, random forest, support vector regression, XGBoost, LASSO regression and ensemble method. Comput Methods Programs Biomed. 2020 Oct;195:105536.

9 Han Z, Zhang Z, Yang X, Li Z, Sang S, Islam MT, Guo AA, Li Z, Wang X, Wang J, Zhang T, Sun Z, Yu L, Wang W, Xiong W, Li G, Jiang Y. Development and interpretation of a pathomics-driven ensemble model for predicting the response to immunotherapy in gastric cancer. J Immunother Cancer. 2024 May;12(5):e008927.

10 Yang S, Fu C, Lian X, Dong X, Zhang Z. Understanding Human-Virus Protein-Protein Interactions Using a Human Protein Complex-Based Analysis Framework. mSystems. 2019 Apr;4(2):e00303-18.

11 Lundberg SM, Lee S-I. A unified approach to interpreting model predictions. In: Lundberg SM, Lee S-I. Proceedings of the 31st International Conference on Neural Information Processing Systems. Red Hook, NY, USA: Curran Associates Inc.; 2017; pp 4768–77.

12 Jiang F, Jiang Y, Zhi H, Dong Y, Li H, Ma S, Wang Y, Dong Q, Shen H, Wang Y. Artificial intelligence in healthcare: past, present and future. Stroke Vasc Neurol. 2017 Dec;2(4):230–43.

13 Deo RC. Machine Learning in Medicine: Will This Time Be Different? Circulation. 2020 Oct;142(16):1521–3.

14 Angraal S, Mortazavi BJ, Gupta A, Khera R, Ahmad T, Desai NR, Jacoby DL, Masoudi FA, Spertus JA, Krumholz HM. Machine learning prediction of mortality and hospitalization in heart failure with preserved ejection fraction. JACC Heart fail. 2020 Jan;8(1):12–21.
